# STM-ac4C: a hybrid model for identification of N4-acetylcytidine (ac4C) in human mRNA based on selective kernel convolution, temporal convolutional network, and multi-head self-attention

**DOI:** 10.3389/fgene.2024.1408688

**Published:** 2024-05-30

**Authors:** Mengyue Yi, Fenglin Zhou, Yu Deng

**Affiliations:** School of Information Engineering, Jingdezhen Ceramic University, Jingdezhen, China

**Keywords:** N4-acetylcytidine, selective kernel convolution, temporal convolutional network, multi-head self-attention, deep learning

## Abstract

N4-acetylcysteine (ac4C) is a chemical modification in mRNAs that alters the structure and function of mRNA by adding an acetyl group to the N4 position of cytosine. Researchers have shown that ac4C is closely associated with the occurrence and development of various cancers. Therefore, accurate prediction of ac4C modification sites on human mRNA is crucial for revealing its role in diseases and developing new diagnostic and therapeutic strategies. However, existing deep learning models still have limitations in prediction accuracy and generalization ability, which restrict their effectiveness in handling complex biological sequence data. This paper introduces a deep learning-based model, STM-ac4C, for predicting ac4C modification sites on human mRNA. The model combines the advantages of selective kernel convolution, temporal convolutional networks, and multi-head self-attention mechanisms to effectively extract and integrate multi-level features of RNA sequences, thereby achieving high-precision prediction of ac4C sites. On the independent test dataset, STM-ac4C showed improvements of 1.81%, 3.5%, and 0.37% in accuracy, Matthews correlation coefficient, and area under the curve, respectively, compared to the existing state-of-the-art technologies. Moreover, its performance on additional balanced and imbalanced datasets also confirmed the model’s robustness and generalization ability. Various experimental results indicate that STM-ac4C outperforms existing methods in predictive performance. In summary, STM-ac4C excels in predicting ac4C modification sites on human mRNA, providing a powerful new tool for a deeper understanding of the biological significance of mRNA modifications and cancer treatment. Additionally, the model reveals key sequence features that influence the prediction of ac4C sites through sequence region impact analysis, offering new perspectives for future research. The source code and experimental data are available at https://github.com/ymy12341/STM-ac4C.

## 1 Introduction

Since the first discovery of RNA chemical modifications 60 years ago, more than 170 types of RNA modifications have been characterized (Zhang Y. et al., 2022). These modifications mainly exist in non-coding RNAs, such as ribosomal RNA (rRNA), transfer RNA (tRNA), and small nuclear RNA (snRNA) (Shi et al., 2020). They are essential for correctly translating the eukaryotic genome and can regulate the same RNA transcript to produce different mRNA and protein products (Schaefer et al., 2017; Cui et al., 2022). Among them, n4-acetylcytidine (ac4C) is a common modification that widely regulates various aspects of RNA metabolism (Karthiya et al., 2020). ac4C mainly occurs in the coding region of mRNA and is catalyzed by the NAT10 enzyme (Jiang et al., 2023; Hu et al., 2024). ac4C modification can affect mRNA’s stability and translation efficiency, and its mechanism is to change the structure and interaction of mRNA and enhance its affinity with ribosome, tRNA, protein, and other RNA (Jin et al., 2020). There have been studies that have found that ac4C plays a role in DNA damage repair, and there have been studies that have found that ac4C is related to the occurrence and development of various diseases, such as cancer, neurodegenerative diseases, viral infections, etc., (Liu et al., 2022; Luo et al., 2023). Therefore, predicting the sites of ac4C is of great significance for understanding its function and designing therapeutic strategies.

In recent years, with the development of high-throughput sequencing technology, Arango et al. used acRIP-seq technology to perform ac4c-specific RNA immunoprecipitation analysis on the transcriptome of human HeLa cells (Arango et al., 2018; 2019). They found more than 4,000 regions containing ac4C. However, acRIP-seq technology cannot accurately locate the position of each ac4C and can only give a rough range. They found that ac4C is widely present in the human transcriptome and is mainly concentrated in the coding regions. They also found that ac4c-modified mRNA is more stable than other mRNA, and its translation level significantly increases after it is acetylated. Because detecting ac4C in mRNA by experimental methods is time-consuming and labor-intensive, computational methods are needed to identify the position of ac4C efficiently and reliably. Currently, some computational methods have been proposed (Zhao et al., 2019; Alam et al., 2020; Wang et al., 2021; Zhang G. et al., 2022; Iqbal et al., 2022; Jia et al., 2023a; 2023c; 2023b; Su et al., 2023; Li et al., 2024).

For example, Zhao et al. developed PACES(Zhao et al., 2019), the first model to use a combination of two random forest (RF) classifiers to predict ac4C sites in human mRNA, using position-specific dinucleotide sequence spectrum (PSDSP) and k-nucleotide frequency (KNF) as features for the two classifiers. Subsequently, Alam et al. developed XG-ac4C (Alam et al., 2020), a model that improved the accuracy of predicting ac4C sites by using the extreme gradient boosting (XGboost) algorithm, combining electron-ion interaction pseudopotentials (EIIP) and electron-ion interaction pseudopotentials of trinucleotide (PseEIIP). Wang et al. designed a model named DeepAc4C(Wang et al., 2021) based on a convolutional neural network (CNN), which used a hybrid feature composed of physicochemical mode and nucleic acid distribution representation and showed better performance in predicting ac4C sites. At the same time, Iqbal et al. proposed a CNN-based deep learning model, DL-ac4C (Iqbal et al., 2022), and compared it with traditional machine learning methods, regression, and Support Vector Machines. Su et al. established a high-quality benchmark dataset and developed a new predictor, iRNA-ac4C (Su et al., 2023). The predictor was based on a gradient boosting decision tree (GBDT), integrating k-mer nucleotide composition, nucleotide chemical property (NCP), and accumulated nucleotide frequency (ANF) as three feature extraction methods, and surpassed the previous models in identifying ac4C sites. Lai and Gao et al. proposed LSA-ac4C (Lai and Gao, 2023), a model that used a two-layer LSTM neural network to learn the dependency relationship of mRNA sequences and used the self-attention mechanism to pay attention to the importance of nucleotides in mRNA sequences. They also trained a transformer-based deep learning model using automated machine learning (AutoML) technology called Auto-ac4C. This model also performed better than the current state-of-the-art (SOTA) models, indicating that it is a reliable baseline and the possibility of further improving the accuracy of ac4C site prediction using deep learning methods. Recently, Jia et al. proposed a deep learning-based prediction model, DLC-ac4C (Jia et al., 2023a). The model uses a variety of features, including three feature encoding schemes, one-dimensional convolutional layers, densely connected convolutional networks (DenseNet), bidirectional long short-term memory networks (Bi-LSTM), channel attention mechanism, and ensemble learning strategy, to capture hidden information features from the sequence perspective. Following DLC-ac4C, Li et al. proposed a deep learning-based multi-module framework, MetaAc4C, which combines pre-trained Bidirectional Encoder Representations from Transformers (BERT) and a Generative Adversarial Network WGAN-GP, where WGAN-GP is used to enhance training data and address data imbalance issues. The core is a Bidirectional Long Short-Term Memory network (BLSTM), which improves model performance through attention mechanisms and residual connections.

Although these models have made some progress, there is still room for improvement in the prediction accuracy of the sites. Therefore, we propose a deep learning-based model named STM-ac4C for identifying N4-acetylcytidine (ac4C) modification sites on human mRNA. The main contributions of this paper are as follows:

1) An innovative hybrid neural network architecture is proposed, which combines the advantages of selective kernel convolution (SKC) (Li et al., 2019), temporal convolutional networks (TCN) (Bai et al., 2018; Raza et al., 2023), and multi-head self-attention mechanisms (MHSA) (Vaswani et al., 2017). This design enables the model to adaptively capture k-mer features of varying lengths, enhances the ability to capture long-term dependencies in mRNA sequences, and achieves adaptive fusion of features, thereby improving the accuracy of predictions and the model’s generalization ability.
2) In terms of feature encoding, this study adopts one-hot encoding (Cheng et al., 2021) to maintain the complete information of the input sequence and avoid information loss. Compared to embedding encoding, one-hot encoding is simple, easy to use, stable, and has no significant impact on model performance while reducing computational resource consumption.
3) The results of ten-fold cross-validation and independent testing show that STM-ac4C outperforms existing models in accuracy, Matthews correlation coefficient, and area under the curve, providing valuable tools and new benchmarks for the related research field.
4) The robustness and generalization ability of the model has been validated on additional balanced and unbalanced datasets, confirming that STM-ac4C maintains stable predictive performance on more challenging datasets.
5) Key sequence features were revealed through sequence region impact analysis, providing a new perspective for understanding the biological significance of mRNA modifications.

In summary, the STM-ac4C model excels in predicting ac4C modification sites on human mRNA, offering a powerful new tool for a deeper understanding of the biological significance of mRNA modifications and cancer treatment.

This paper is divided into the following sections: Section 1 serves as the introduction, presenting the research background, significance, related work, and main contributions. Section 2 elaborates on the research methods, including the selection of datasets, data preprocessing steps, feature encoding techniques, model architecture design details, and performance evaluation criteria. Section 3 displays the experimental results and in-depth analysis, covering the classification performance on benchmark datasets, model comparative analysis, ablation experiments, comparisons with existing technologies, importance assessment of sequence regions, and robustness tests. Section 4 is the conclusion, summarizing the research findings, discussing the study’s limitations, and looking forward to future research directions.

## 2 Materials and methods

### 2.1 Dataset and data preprocessing

We used the high-quality dataset constructed by Su et al. to evaluate our model. The dataset was derived from the experimental data of Arango et al. (Arango et al., 2018), which identified 4,250 ac4C peaks using the acRIP-seq method (Zhang X. et al., 2022). Su et al. (Su et al., 2023) extracted sequences of 100 nt up and down as positive samples to create a reliable dataset, starting from the cytosine near each ac4C peak. They also randomly sampled 201 nt sequences centered on cytosines from non-peak regions as negative samples. They used the CD-HIT (Fu et al., 2012) tool with a threshold of 0.8 to remove redundant sequences. To balance the dataset, they selected 2,758 sequences from each positive and negative sample. Finally, they randomly divided the positive and negative data into training and independent test sets at 4:1. The training set contained 2,206 positive and 2,206 negative samples, and the test set contained 552 positive and 552 negative samples. The details of the dataset are shown in Table 1.

**TABLE 1:** Statistics of the baseline dataset.

Original	Training	Testing
Positive	2,206	552
Negative	2,206	552
Total	4,412	1,104

### 2.2 Preprocessing

#### 2.2.1 Feature encoding method

One-hot encoding (Cheng et al., 2021) is a data preprocessing and feature engineering method that can transform discrete categorical variables into sparse binary codes. This method has wide applications in machine learning, deep learning, bioinformatics, and other fields. The principle of one-hot encoding is given a variable with n categories to generate a new feature vector of length n, where only one element is 1, and the rest are 0. The position of the one indicates the category of the variable. For example, if there are three categories, A, B, and C, their one-hot encodings are $\left(1,0,0\right)$ , $\left(0,1,0\right)$ , and $\left(0,0,1\right)$ respectively.

The advantage of one-hot encoding is that each category has an independent binary representation, and these vectors are mutually orthogonal, meaning that their inner product is zero, and there is no size relationship. This can avoid using ordered or continuous values to represent class labels because this would cause problems in calculations such as weight matrices or distance metrics.

We can use one-hot encoding to represent nucleotides in RNA sequences. RNA sequences are composed of four nucleotides, A, U, G, and C, which correspond to different bases. If the type of nucleotide is unknown, use N to represent it. The following formula can be used to convert them into one-hot encoding:

$$\begin{array}{c} A\;\to \left(1,0,0,0,0\right)\\ U\;\to \left(0,1,0,0,0\right)\\ G\;\to \left(0,0,1,0,0\right)\\ C\;\to \left(0,0,0,1,0\right)\\ N\;\to \left(0,0,0,0,1\right) \end{array}$$ (1)

**FIGURE 1:**
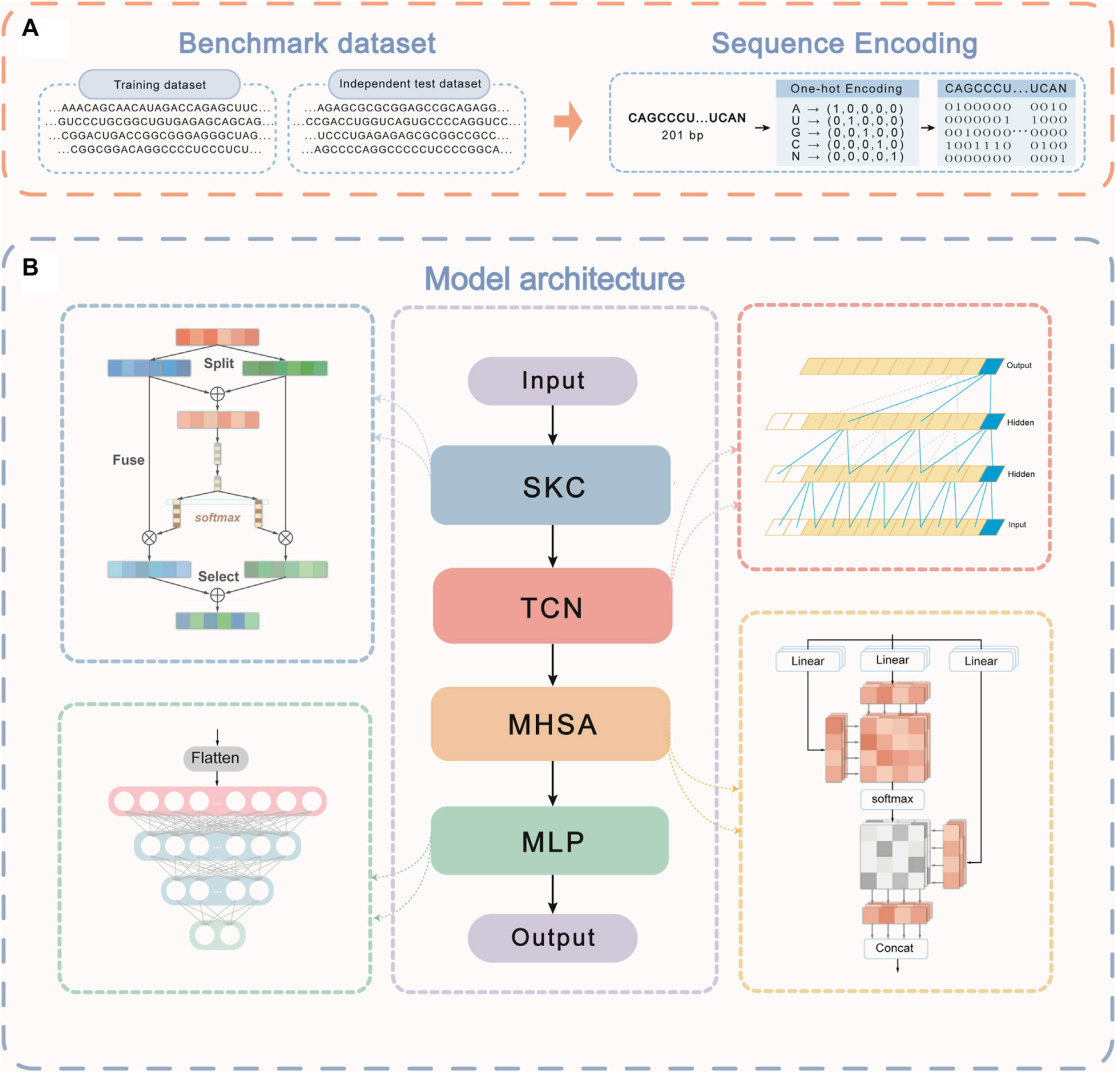
The schematic diagram of STM-ac4C. **(A)** Feature encoding. One-hot encoding converts the 201 nt RNA sequence into a 5 × 201 matrix. **(B)** Model architecture. The encoded feature matrix is sequentially input to three modules: selective kernel convolution (SKC), temporal convolution network (TCN), and multi-head self-attention (MHSA) for feature extraction. Then, the extracted feature matrix is flattened into a one-dimensional vector, and finally, an MLP layer is used for binary classification to predict whether the sequence contains ac4C modification sites.

As shown in Figure 1A, this way, an RNA sequence of length 201 bp, can be converted into a 5 x 201 matrix by one-hot encoding, where each column represents a nucleotide, and each row represents a feature.

### 2.3 Model architecture

In biology, the analytical and predictive capabilities of AI models have been empirically demonstrated across multiple research directions (Ukwuoma et al., 2022b; 2022a; 2023; Fazmiya et al., 2024; Heyat et al., 2024; Sumbul et al., 2024). Based on this, we propose an innovative hybrid neural network model, STM-ac4C, designed to accurately identify N4-acetylcytidine (ac4C) modification sites on human mRNA. As illustrated in Figure 1B, the model skillfully integrates Selective Kernel Convolution (SKC), Temporal Convolutional Network (TCN), and Multi-Head Self-Attention (MHSA) mechanisms. This combination enables STM-ac4C to efficiently capture diverse patterns, structures, and correlations in RNA sequences, which significantly improves prediction accuracy and robustness. SKC overcomes the limitations of traditional fixed kernel sizes by adaptively capturing k-mer features of varying lengths. Compared with LSTM, TCN performs better in modeling long-term dependencies and can effectively handle the temporal and dynamic changes of RNA sequences. The MHSA mechanism, on the other hand, achieves in-depth capture of global and local correlations of RNA sequences by enhancing the model’s characterization capability. At the end of the model, a Multi-Layer Perceptron (MLP) serves as the classifier, transforming the output from the previous layer into the final prediction results using nonlinear activation functions and fully connected layers.

#### 2.3.1 Selective kernel convolution

Selective Kernel Convolution (SKC) (Li et al., 2019) is an innovative convolutional method proposed by Li et al., in 2019. It is designed to simulate the dynamic adaptability of human visual neurons’ receptive fields. SKC intelligently selects the most suitable kernel size by analyzing the multi-scale information of the input features, thereby adjusting the range of the neurons’ receptive fields. This method can be seamlessly integrated into existing convolutional network architectures, significantly enhancing the network’s performance and efficiency while maintaining lightweight parameters and computation.

The core advantage of SKC lies in its subtle improvement over traditional convolutional networks—achieving the effect of multi-size convolutional kernels through grouped and dilated convolutions, thus only slightly increasing the parameters and computational load. The operation process of SKC includes three key steps: split, fuse, and select. In the split phase, the input feature map is convolved with convolutional kernels of different sizes, producing multiple feature sub-maps representing different receptive field sizes. These feature sub-maps are comprehensively integrated during the fusion phase to form a global feature representation containing selection weight information. Finally, based on the calculated selection weights, different-sized feature sub-maps are weighted and aggregated in the selection phase to output the final feature map.

**FIGURE 2:**
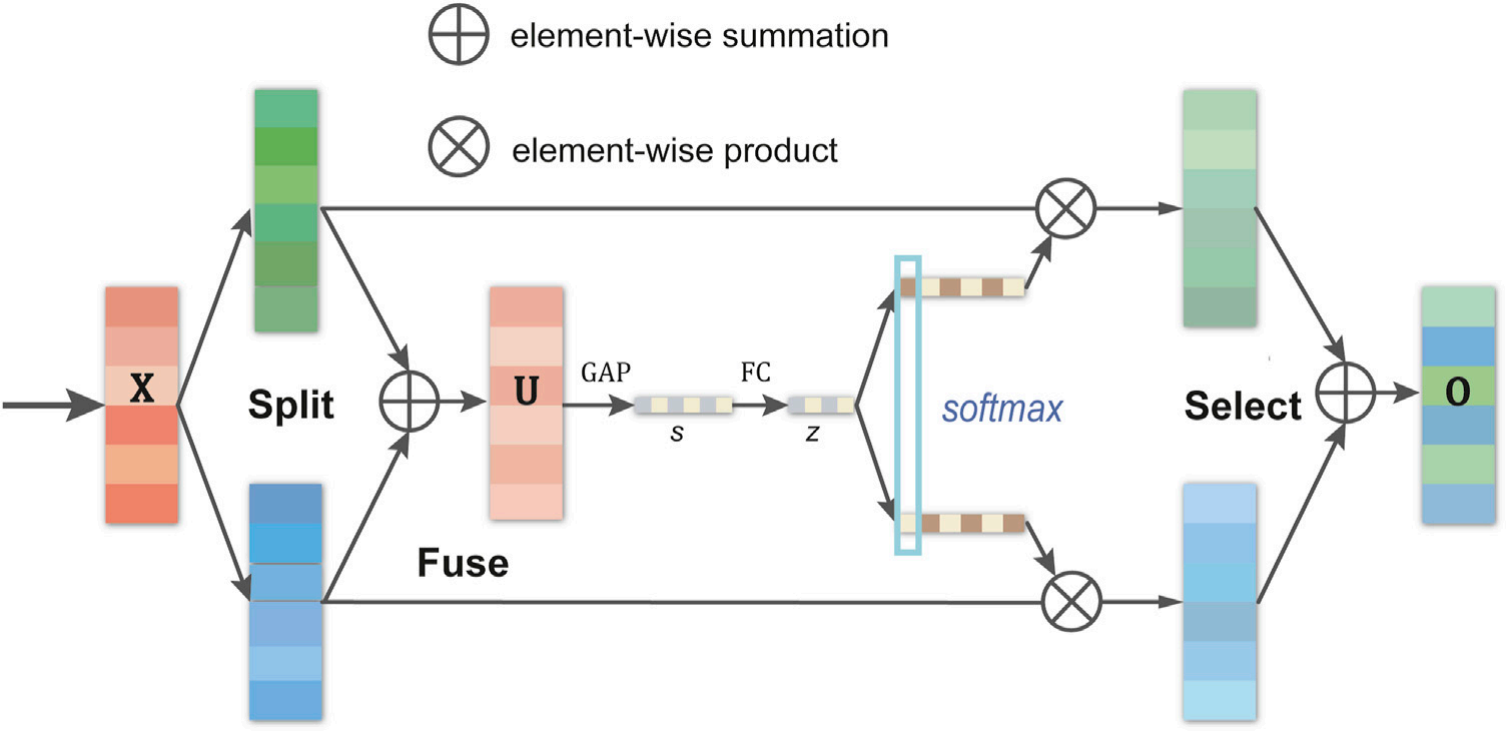
The structure of selective kernel convolution.

Figure 2 shows the structure of SKC. To further elucidate the mathematical model of SKC, we introduce the following formulas to describe its operation process:

1) *Split*: For a given input feature map $X\in R^{C_{in}\times L}$ , where $C_{in}$ represents the number of input channels, and L represents the input length. This feature map is processed by a series of convolutional kernels $K_{i}$ (a total of M ), applying the ReLU activation function and Norm normalization method, generating multiple feature sub-maps $U_{i}\in R^{C_{out}\times L}$ :

$$U_{i}=ReLU\left(Norm\left(K_{i}*X\right)\right)$$ (2)

2) *Fuse*: Through element-wise addition, all feature sub-maps $U_{i}$ are fused into a global feature map U :

$$U=\sum_{i=0}^{M-1}U_{i}$$ (3)

3) *Select*: First, a global descriptor s is generated on the global feature map U by applying Global Average Pooling (GAP):

$$s=GAP\left(U\right)$$ (4)

Then, the descriptor s is transformed into an attention vector $z\in R^{M\cdot C_{out}}$ through two fully connected layers ( FC ), where the first FC layer uses the ReLU activation function. The second FC layer does not use an activation function:

$$z=FC\left(ReLU\left(FC\left(s\right)\right)\right)$$ (5)

Next, for each channel c , the softmax of z is computed to obtain the attention weights $A_{m,c}$ , where $z_{m,c}$ represents the m+M⋅c th element of vector z .

$$A_{m,c}=\frac{e^{z_{m,c}}}{\sum_{j=0}^{M-1}e^{z_{j,c}}}$$ (6)

Finally, using the attention weights $A_{m,c}$ , each feature sub-map $U_{m,c}$ is weighted and summed for each channel to obtain the c -th channel of the output feature map $O_{c}$ , and the final output O is:

$$O_{c}=\sum_{m=0}^{M-1}A_{m,c}\cdot U_{m,c}$$ (7)

$$O=\left[O_{0},O_{1},\ldots ,O_{C_{out-1}}\right]$$ (8)

#### 2.3.2 Temporal convolutional network

Temporal Convolutional Networks (TCN) (Bai et al., 2018; Raza et al., 2023) are network modules for modeling and forecasting sequential data. Their design is inspired by Deep Convolutional Neural Networks (DCNN) (Gu et al., 2018; Abbas et al., 2021), with improvements made to better handle sequential tasks. A significant advantage of TCN is its ability to compute outputs in parallel, significantly enhancing computational efficiency and reducing the computational burden and memory requirements compared to Recurrent Neural Networks (RNN) (Pascanu et al., 2013).

TCN effectively processes time-series data using causal convolutions, ensuring stability and reliability when handling historical information. Causal convolution is a special type only related to the current or previous inputs. It maintains the consistency of the input and output sequence lengths through zero-padding techniques, thereby solving the problem of recursive dependencies. The output of a causal convolution, $y_{t}$ , can be calculated using the following formula:

$$y_{t}=\sum_{i=0}^{k-1}w_{i}\cdot x_{t-i}+b$$ (9)

Where $w_{i}$ represents the weights of the convolutional kernel, b is the bias, and k is the size of the convolutional kernel.

To enhance model performance, TCN also employs dilated convolutions and residual connections (He et al., 2016). Dilated convolutions expand the receptive field by increasing the stride of the convolutional kernel, allowing it to capture longer-range dependencies. Residual connections enhance information flow by introducing skip connections, preventing gradient vanishing or explosion issues. The output of a causal dilated convolution, $y_{t}^{\prime }$ , can be represented by the following formula:

$$y_{t}^{\prime }=\sum_{i=0}^{k-1}w_{i}\cdot x_{t-d\cdot i}+b$$ (10)

Here, d is the dilation factor, which determines the interval at which the convolutional kernel covers the input sequence. When d=1 , the causal dilated convolution degenerates into a standard causal convolution. A larger dilation factor allows the top-level output to represent a broader range of inputs, effectively expanding the receptive field of the convolutional network.

**FIGURE 3:**
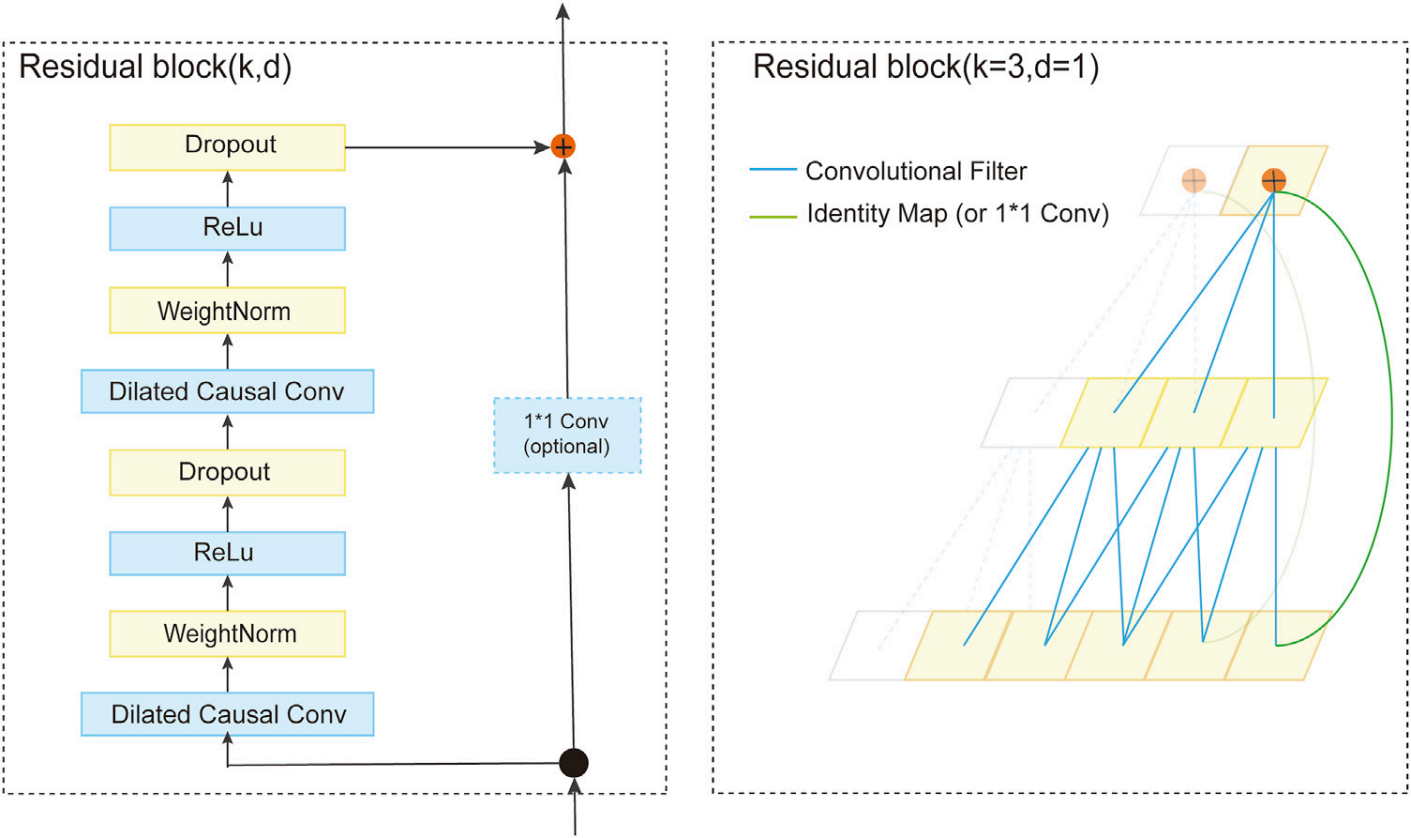
The architecture of the residual block.

A TCN comprises a series of residual blocks containing two layers of dilated causal convolution and ReLU activation function. Weight normalization is applied to the convolutions for stability and efficiency. As depicted in Figure 3, the architecture of a residual block is defined. The TCN processes the input sequence via these residual blocks. The convolutional kernel size, denoted as k , is constant across the network. In contrast, the dilation factor, represented as d, increases exponentially relative to the network’s depth. This design ensures comprehensive coverage of each input point by at least one filter, thereby extending the effective historical range without compromising the depth of the network.

#### 2.3.3 Multi-head self-attention

**FIGURE 4:**
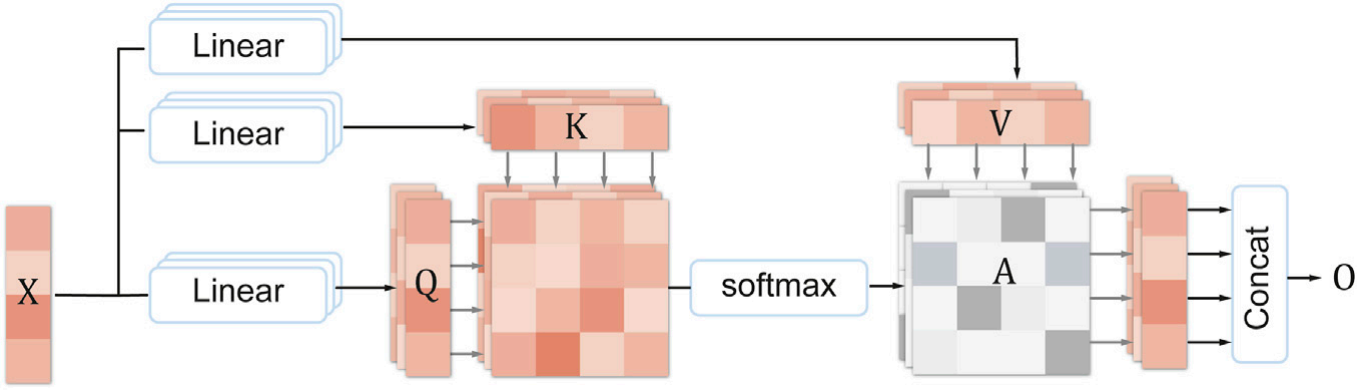
The structure of multi-head self-attention.

Multi-head self-attention (MHSA) (Vaswani et al., 2017) is an improved version of self-attention, which uses multiple attention heads to process different positions and features of the input sequence, thereby enhancing the model’s feature extraction and interpretability performance on sequential data. Specifically, each attention head multiplies the elements of the input sequence (such as words or pixels) with three matrices, respectively, to obtain three vectors: query, key, and value. Then, according to the similarity between the query and the key, the value vectors are weighted and summed to obtain a sub-representation of the output sequence. Finally, the sub-representations of all attention heads are concatenated to obtain the final representation of the output sequence. This way, the model can flexibly focus on the important parts of the input sequence, thus effectively extracting and utilizing the key information in the sequence. The specific formula of multi-head self-attention is as follows:

Figure 4 shows the structure of MHSA. Assume that the input sequence is $X=\left(x_{1},x_{2},\ldots ,x_{n}\right)$ , where $x_{i}\in R^{d}$ is the feature vector of the i -th element, and d is the feature dimension. Multi-head self-attention uses h different matrices $W_{q}^{i},W_{k}^{i},W_{v}^{i}\in R^{d\times d_{k}}$ , where i=1,2,…,h , and $d_{k}$ is the feature dimension of each head, satisfying $hd_{k}=d$ . For each head i , the query, key, and value vectors are calculated as follows:

$$\begin{array}{c} Q^{i}=XW_{q}^{i}\in R^{n\times d_{k}}\\ K^{i}=XW_{k}^{i}\in R^{n\times d_{k}}\\ V^{i}=XW_{v}^{i}\in R^{n\times d_{k}} \end{array}$$ (11)

Then, the attention weight matrix $A^{i}\in R^{n\times n}$ of each head is calculated, where $A_{jk}^{i}$ represents the attention weight of the j -th position to the k -th position, as follows:

$$A^{i}=\text{softmax}\left(\frac{Q^{i}{\left(K^{i}\right)}^{T}}{\sqrt{d_{k}}}\right)$$ (12)

Where softmax is performed row-wise, and $\sqrt{d_{k}}$ is a scaling factor that balances the influence of different dimensions. Finally, the output sequence $O^{i}\in R^{n\times d_{k}}$ of each head is calculated, and they are concatenated column-wise to obtain the final output sequence $O\in R^{n\times d}$ , as follows:

$$\begin{array}{l} O^{i}=A^{i}V^{i}\\ O=\text{concat}\left(O^{1},O^{2},\ldots ,O^{h}\right) \end{array}$$ (13)

### 2.4 Performance evaluation

In this study, we selected five common evaluation metrics for machine learning classification prediction tasks to evaluate the predictive performance of the model, namely, sensitivity (Sn), specificity (Sp), accuracy (Acc), Matthews correlation coefficient (MCC), and area under the receiver operating characteristic curve (AUC) (Sokolova and Lapalme, 2009). The formulas for these metrics are as follows:

$$\begin{array}{l} Sn=\frac{TP}{TP+FN}\\ Sp=\frac{TN}{TN+FP}\\ Acc=\frac{TP+TN}{TP+TN+FP+FN}\\ MCC=\frac{TP\times TN-FP\times FN}{\sqrt{\left(TP+FN\right)\times \left(TN+FN\right)\times \left(TP+FP\right)\times \left(TN+FP\right)}}\\ AUC=\int_{0}^{1}Sn\left(FP\right)dFP \end{array}$$ (14)

Where TP, FP, TN, and FN represent the number of true positives, false positives, true negatives, and false negatives, respectively. They are the elements of the confusion matrix constructed based on the model’s prediction results and the true labels. AUC is the area under the receiver operating characteristic curve (ROC curve), which is a curve drawn with FP as the horizontal axis and Sn as the vertical axis, reflecting the changes in sensitivity and specificity of the model at different thresholds. Generally speaking, the higher the values of these metrics, the better the model’s predictive performance.

The STM-ac4C was implemented in a Python 3.9.16 environment, utilizing the torch 2.0.0+cu118 framework. The model training employed an Adam optimizer, with a learning rate set to 1e-4, a batch size of 64, and a maximum training epoch set at 300. The loss function chosen was binary cross-entropy. Early stopping was also adopted to prevent overfitting, meaning the training would be halted if the $0.5\times \left(MCC+AUC\right)$ on the validation set did not improve over 20 consecutive epochs.

## 3 Result and discussion

### 3.1 Classification performance on the benchmark dataset

**FIGURE 5:**
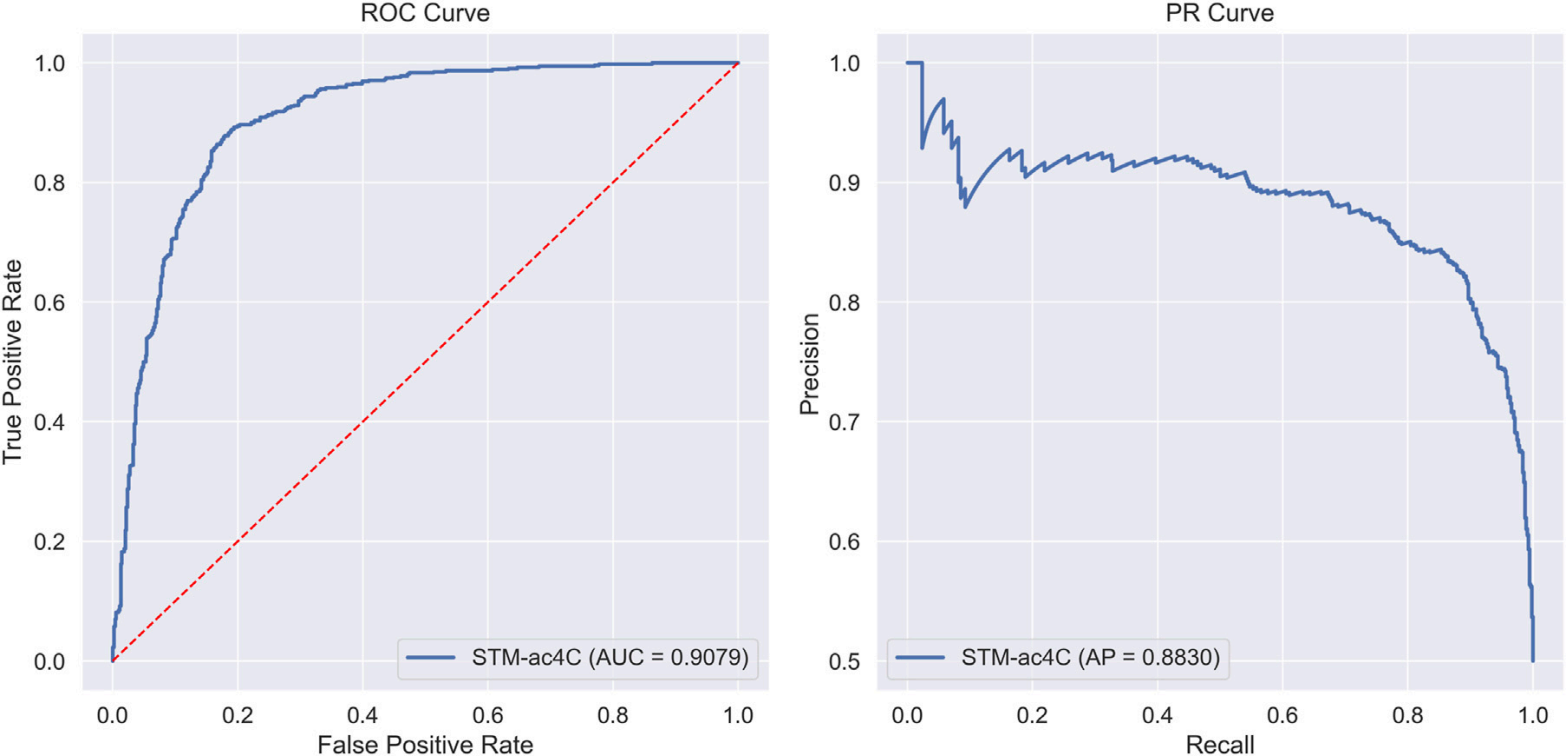
ROC and PR curves of STM-ac4C on the independent test dataset.

In this section, we evaluated the classification performance of the STM-ac4C model on the benchmark dataset. We meticulously recorded the model’s performance in each fold using ten-fold cross-validation. The evaluation metrics include Sensitivity (Sn), Specificity (Sp), Accuracy (Acc), Matthews Correlation Coefficient (MCC), and Area Under the Curve (AUC), with specific data presented in Table 2. The model’s average values and standard deviations for each metric are as follows: Acc is 82.57% ± 1.54%, Sn is 86.49% ± 3.76%, Sp is 78.65% ± 2.36%, MCC is 65.43% ± 2.89%, and AUC is 88.52% ± 1.02%. On the independent test set, the model demonstrated superior performance, with an Acc of 84.78%, Sn of 85.87%, Sp of 83.7%, MCC of 69.58%, and AUC of 90.79%. Both cross-validation and independent testing have demonstrated that the sensitivity (Sn) surpasses the specificity (Sp), signifying that the STM-ac4C model exhibits a pronounced superiority in identifying positive samples. Figure 5 intuitively displays the model’s performance through ROC and PR curves, where the ROC curve reflects the relationship between true positive rate and false positive rate at different thresholds, and the PR curve shows the relationship between precision and recall.

**TABLE 2:** Classification results of STM-ac4C on each fold.

Fold number	Sn (%)	Sp (%)	Acc (%)	MCC (%)	AUC (%)
0	90.95	69.68	80.32	62.05	86.88
1	85.97	77.38	81.67	63.58	87.04
2	84.16	79.55	81.86	63.78	89.74
3	82.35	80.91	81.63	63.27	88.67
4	87.78	77.27	82.54	65.43	88.58
5	85.07	79.09	82.09	64.28	88.49
6	86.36	81.45	83.90	67.89	90.02
7	88.18	83.71	85.94	71.96	89.22
8	86.82	80.09	83.45	67.05	88.54
9	87.27	77.38	82.31	64.96	88.01

### 3.2 Comparison of using one-hot encoding, NCP encoding and embedding encoding

**FIGURE 6:**
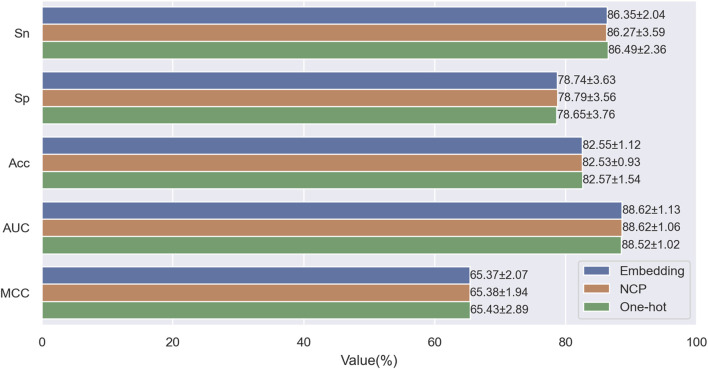
Performance comparison of One-hot encoding with NCP encoding and Embedding encoding on the training dataset.

When encoding sequence features, we aim to preserve as much information as possible from the input sequence while avoiding information loss or redundancy caused by manual feature extraction. To this end, we only considered three encoding schemes: One-hot encoding, NCP encoding (Chen et al., 2016; 2017; Nguyen-Vo et al., 2019), and Embedding encoding (Hasan et al., 2020; Jin et al., 2022b; Lai and Gao, 2023). One-hot encoding is widely used in machine learning, deep learning, bioinformatics, and other fields due to its simplicity, ease of use, stability, and other advantages. NCP encoding converts nucleotide sequences into numerical vectors, which consider nucleotides’ chemical properties and binding characteristics, thereby improving the efficiency and accuracy of sequence analysis. Embedding encoding is a neural network-based method that encodes each categorical element in the sequence into a learnable vector and updates it continuously during the training process, thus learning the semantics and context information of the elements, effectively compressing and abstracting features. To evaluate the impact of the three encoding schemes on model performance, we performed ten-fold cross-validation on the training set, using different encoding methods as model inputs. Figure 6 shows the evaluation metrics under different encoding schemes. From the figure, we can see no significant difference between the results of the three encoding methods. However, embedding encoding increases the model parameters and computational complexity, requiring more time and computational resources during training. In contrast, the one-hot encoding scheme has the highest accuracy (Acc) and Matthews correlation coefficient (MCC): 82.57% and 65.43%. Considering all aspects, we chose one-hot encoding as the final encoding scheme.

### 3.3 Comparison of using TCN, LSTM, GRU and transformer

**FIGURE 7:**
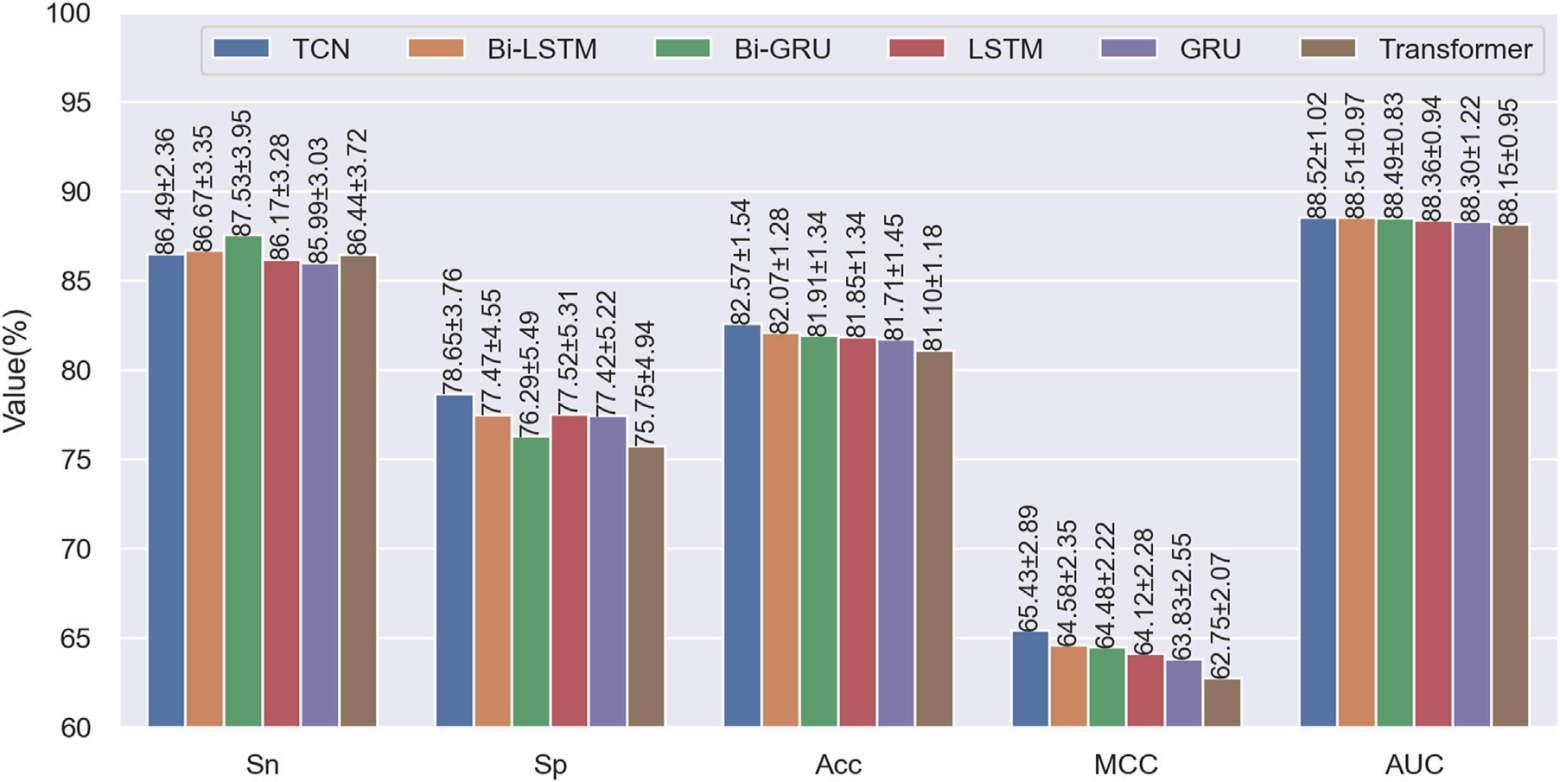
Performance comparison using TCN, LSTM, GRU, and Transformer on the training dataset.

In STM-ac4C, TCN is mainly used to model the long-term dependencies in sequences. In contrast, some of the current sequence modeling methods are mainly based on variants of RNN, such as long short-term memory networks (LSTM) (Cheng et al., 2021; Zhang G. et al., 2022), gated recurrent units (GRU) (Jin et al., 2022b; Nguyen-Vo et al., 2023; Sultana et al., 2023), or Transformer (Jin et al., 2022a; Tsukiyama et al., 2022; Zeng et al., 2023) network modules based on self-attention mechanisms to build predictors. Although these modules have advantages in processing sequence data, they still have limitations. LSTM and GRU can effectively handle short-term dependencies, but they have difficulty capturing long-term dependencies and are prone to gradient vanishing or exploding problems. Transformer uses self-attention mechanisms and position encoding, which can calculate the dependencies between any positions in the sequence, thus solving the problem of long-term dependencies. However, the over-parameterization of the transformer makes it time-consuming and easy to overfit when dealing with small data sets. To verify the advantages of TCN, we replaced TCN with Transformer and LSTM, GRU, and their bidirectional forms in the model framework of STM-ac4C, built multiple different predictors, and evaluated them on the training set using a ten-fold cross-validation method, and compared their performance on various evaluation indicators. As shown in Figure 7, TCN outperforms other modules on most evaluation indicators, indicating that TCN can better capture the long-term dependencies in the sequence, thus improving prediction accuracy. Therefore, we choose TCN as the core module of the STM-ac4C model to improve the model’s prediction performance.

### 3.4 Ablation experiments for STM-ac4C

**FIGURE 8:**
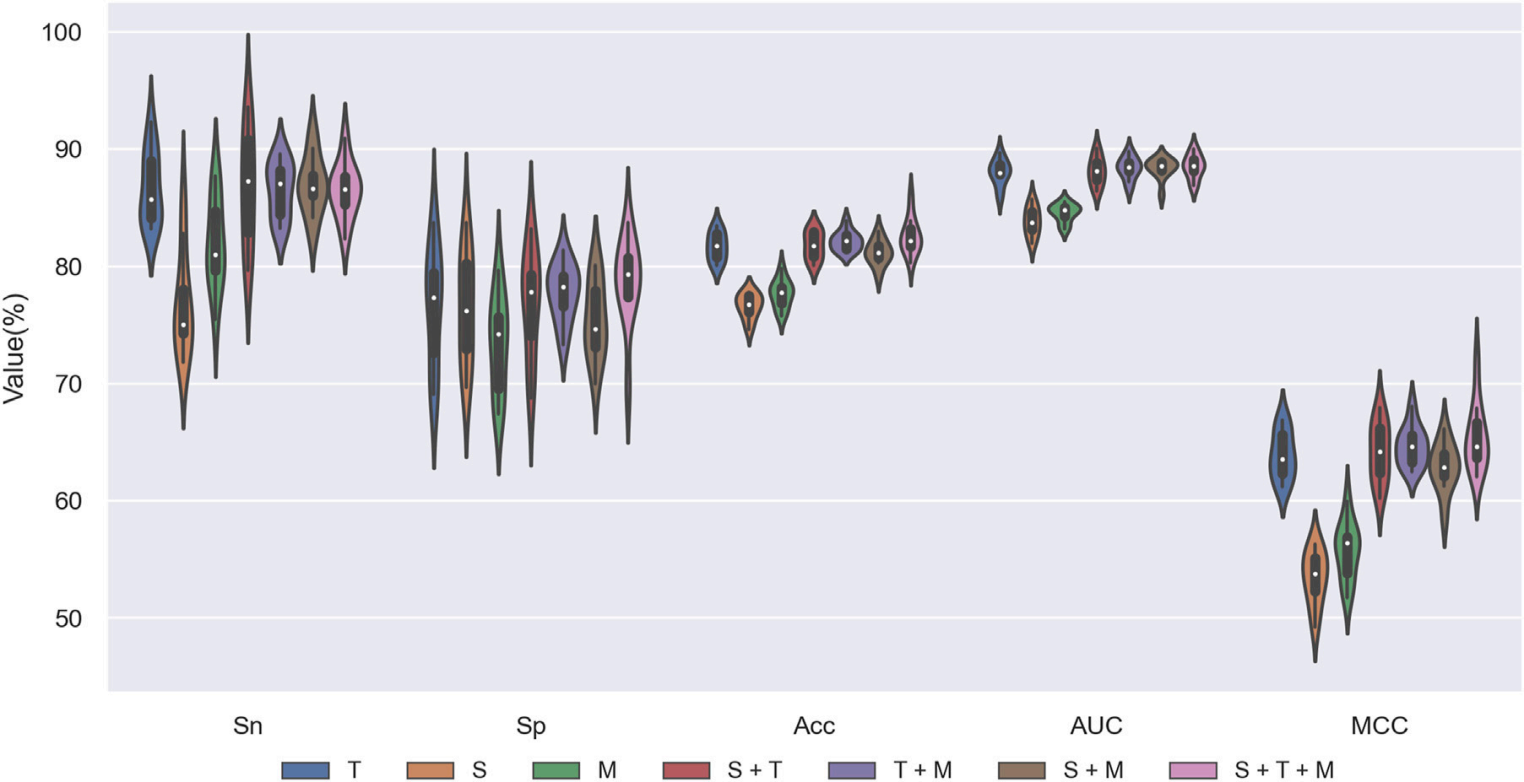
Ablation results of STM-ac4C on the training dataset.

We designed ablation experiments to investigate the contributions of SKC, TCN, and MHSA modules to STM-ac4C. We removed or disabled one or more modules and then performed ten-fold cross-validation on the training set to evaluate the performance of different configurations. Figure 8 and Table 3 show the results of the ablation study, where “√” indicates that the module was used, and “-” indicates that the module was removed or disabled. The table shows that the TCN module has the most significant impact on the model performance, while the SKC and MHSA modules have relatively weaker effects. Using only the TCN module can achieve good performance, with ACC, MCC, and AUC of 81.69%, 63.87%, and 88.01%, respectively. At the same time, each module has some contribution, and removing any module will decrease model performance. When all three modules are used, the model achieves the highest Acc, MCC, and AUC values of 82.57%, 65.57%, and 88.52%. Based on these results, we chose the complete combination of the three modules as the optimal configuration for the STM-ac4C model.

**TABLE 3:** Ablation experiments results for STM-ac4C.

SKC	-	√	-	√	-	√	√
TCN	√	-	-	√	√	-	√
MHSA	-	-	√	-	√	√	√
Sn (%)	86.71 ± 3.15	76.79 ± 4.47	82.00 ± 3.88	86.68 ± 4.89	86.54 ± 2.40	**86.94 ± 2.52**	86.49 ± 2.36
Sp (%)	76.65 ± 5.00	76.48 ± 4.71	73.35 ± 4.08	76.93 ± 4.57	77.79 ± 2.41	75.43 ± 3.34	**78.65 ± 3.76**
Acc (%)	81.69 ± 1.21	76.63 ± 1.07	77.68 ± 1.20	81.80 ± 1.21	82.16 ± 0.84	81.19 ± 1.07	**82.57 ± 1.54**
MCC (%)	63.87 ± 2.05	53.46 ± 2.29	55.71 ± 2.42	64.16 ± 2.51	64.63 ± 1.69	62.88 ± 2.05	**65.43 ± 2.89**
AUC (%)	88.01 ± 1.11	83.83 ± 1.23	84.57 ± 0.76	88.15 ± 1.21	88.40 ± 0.94	88.37 ± 0.89	**88.52 ± 1.02**

The bold font is used to distinctly indicate the highest values achieved for each evaluation metric.

### 3.5 Performance comparison with existing methods

**FIGURE 9:**
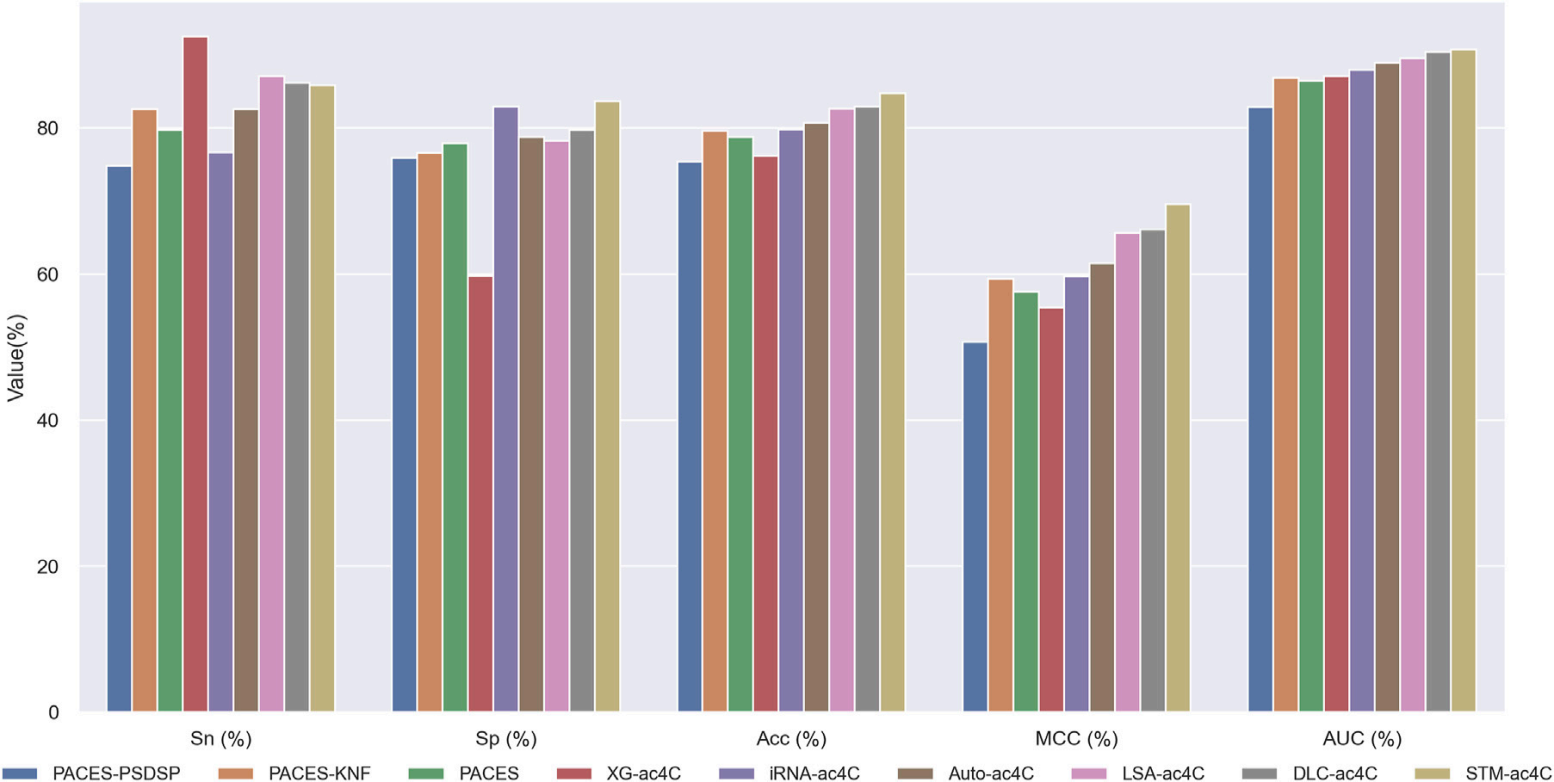
Performance comparison with other predictors on the independent test.

Predicting ac4C sites in human mRNA is a vital bioinformatics problem, and several methods have been proposed and published, such as PACES(Zhao et al., 2019), XG-ac4C (Alam et al., 2020), iRNA-ac4C (Su et al., 2023), DLC-ac4C (Jia et al., 2023a), and LSA-ac4C (Lai and Gao, 2023). However, the performance of these methods is hard to compare directly because they use different datasets and evaluation criteria. In order to evaluate our proposed STM-ac4C method fairly and rigorously, we chose three methods that use the same dataset, iRNA-ac4C, DLC-ac4C, and LSA-ac4C, as comparison objects. We also referred to the results of two methods reported in the LSA-ac4C paper: PACES and XGac4C were retrained using the same dataset, and Auto-ac4C was obtained using the AutoML framework AutoGluon (Erickson et al., 2020; Romero et al., 2022). We compared the performance of these methods on ten-fold cross-validation and independent test sets, and the results are shown in Tables 4 and Table 5. Figure 9 shows the performance comparison on the independent test set. The results indicate that XG-ac4C has very high sensitivity (Sn) in both ten-fold cross-validation and independent test sets, reaching 93.38% and 92.57%, respectively, demonstrating excellent performance in detecting positive samples. However, its specificity (Sp) is relatively low, only 54.76% and 59.78%, which may lead to more false positives when identifying ac4C sequences. In contrast, STM-ac4C surpassed other methods in most evaluation metrics, especially in terms of accuracy (ACC), Matthews correlation coefficient (MCC), and area under the curve (AUC). In ten-fold cross-validation and independent test sets, STM-ac4C’s ACC, MCC, and AUC reached 82.57%, 65.43%, 88.52%, and 84.78%, 69.58%, 90.79%, respectively, all of which are the highest among all models. Particularly in the independent test set, STM-ac4C’s MCC is 3.51% higher than the highest LSA-ac4C. These results suggest that STM-ac4C can effectively balance classification performance and prediction accuracy, effectively identifying ac4C sites in human mRNA.

**TABLE 4:** The performance over the 10-fold cross-validation.

Method	Sn (%)	Sp (%)	Acc (%)	MCC (%)	AUC (%)
PACES-PSDSP	74.57 ± 2.75	72.53 ± 3.85	73.55 ± 1.95	47.18 ± 3.87	80.96 ± 1.61
PACES-KNF	80.51 ± 2.68	74.71 ± 2.89	77.61 ± 1.2	55.38 ± 2.44	85.32 ± 1.2
PACES	78.38 ± 1.86	75.75 ± 2.95	77.06 ± 1.13	54.2 ± 2.19	84.84 ± 1.28
XG-ac4C	**93.38 ± 1.23**	54.76 ± 2.03	74.07 ± 0.87	52.22 ± 1.62	85.24 ± 1.22
iRNA-ac4C	77.02	**83.01**	80.03	60.1	87.5
Auto-ac4C	85.08 ± 4.11	77.01 ± 3.61	81.05 ± 1.58	62.47 ± 3.33	87.97 ± 1.48
LSA-ac4C	85.54 ± 3.17	78.51 ± 3.13	82.03 ± 1.49	64.31 ± 2.94	87.97 ± 1.18
DLC-ac4C	83.19	77.26	80.07	60.64	87.74
STM-ac4C	86.49 ± 3.76	78.65 ± 2.36	**82.57 ± 1.54**	**65.43 ± 2.89**	**88.52 ± 1.02**

The bold font is used to distinctly indicate the highest values achieved for each evaluation metric.

**TABLE 5:** The performance over the independent test.

Method	Sn (%)	Sp (%)	Acc (%)	MCC (%)	AUC (%)
PACES-PSDSP	74.82	75.91	75.36	50.73	82.87
PACES-KNF	82.6	76.63	79.62	59.35	86.92
PACES	79.71	77.9	78.8	57.62	86.48
XG-ac4C	**92.57**	59.78	76.18	55.42	87.13
iRNA-ac4C	76.7	82.91	79.81	59.7	88
Auto-ac4C	82.61	78.8	80.71	61.46	88.94
LSA-ac4C	87.13	78.26	82.7	65.66	89.53
DLC-ac4C	86.23	79.71	82.97	66.08	90.42
STM-ac4C	85.87	**83.7**	**84.78**	**69.58**	**90.79**

The bold font is used to distinctly indicate the highest values achieved for each evaluation metric.

### 3.6 Sequence region impact analysis

We designed and conducted two experiments to better understand the distribution of critical regions within ac4C sequences and their impact on model prediction performance. These experiments aimed to reveal how different sequence regions specifically affect the model’s predictive capabilities and to identify areas critical for prediction.

**FIGURE 10:**
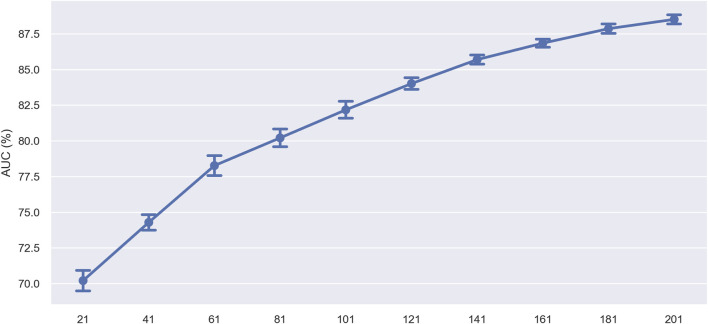
Performance comparison of using different sequence lengths on the training dataset.

In the initial experiment, we evaluated the impact of sequence length on model prediction performance by progressively shortening the sequence length. Specifically, we do this by removing 10 nucleotides from both ends of the sequence each time. To assess the corresponding model’s performance, we performed ten-fold cross-validation on training sets with lengths of 21, 41, 61, 81, 101, 121, 141, 161, 181, and 201 nucleotides (nt). The model’s performance (measured by AUC value) trend with varying sequence lengths is displayed in Figure 10. The results showed that the model’s performance peaked at a sequence length of 201 nucleotides, indicating that this length contained the complete information necessary for accurate prediction. As the sequence length decreased, the model’s performance gradually declined, with an accelerating rate of decline. This may be due to the loss of crucial sequence information, preventing the model from capturing enough features for accurate prediction. Additionally, the accelerated decline in performance may indicate that certain regions within the sequence contribute more significantly to model prediction, possibly containing key biological signals such as specific motifs or structural features crucial for identifying ac4C sites.

**FIGURE 11:**
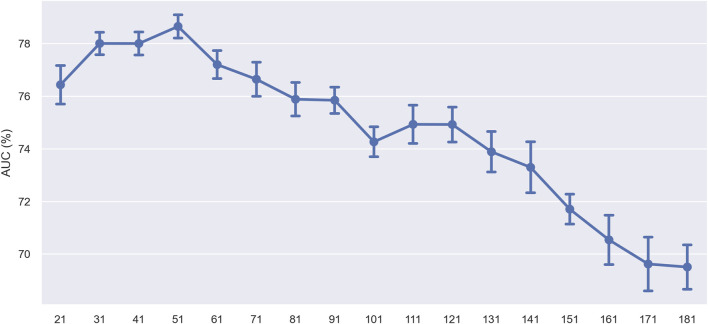
Performance comparison of using different locations of window center points on the training dataset.

In the subsequent experiment, we set a fixed window size of 41 nucleotides (nt). We slid it across the sequence in 10 nt steps to assess the specific impact of different sequence regions on model prediction performance. After each slide, we used only the sequence within the window for ten-fold cross-validation and monitored changes in model performance. Figure 11 presents the experimental results, where the *x*-axis represents the window’s central position in the sequence, and the *y*-axis represents the AUC value. The results showed that the model’s performance fluctuated with the movement of the window’s center point, revealing regions within the sequence crucial for predicting ac4C sites. Overall, the model’s performance showed a decreasing trend, suggesting that the sequence region to the left of the ac4C site, i.e., the 5′direction, plays a crucial role in model prediction. The performance increase observed at position 51 of the sequence suggests that this area may contain specific sequence features essential for identifying ac4C sites. These performance increases may reflect key biological signals that the model can recognize, such as specific nucleotide patterns or structural features.

**FIGURE 12:**
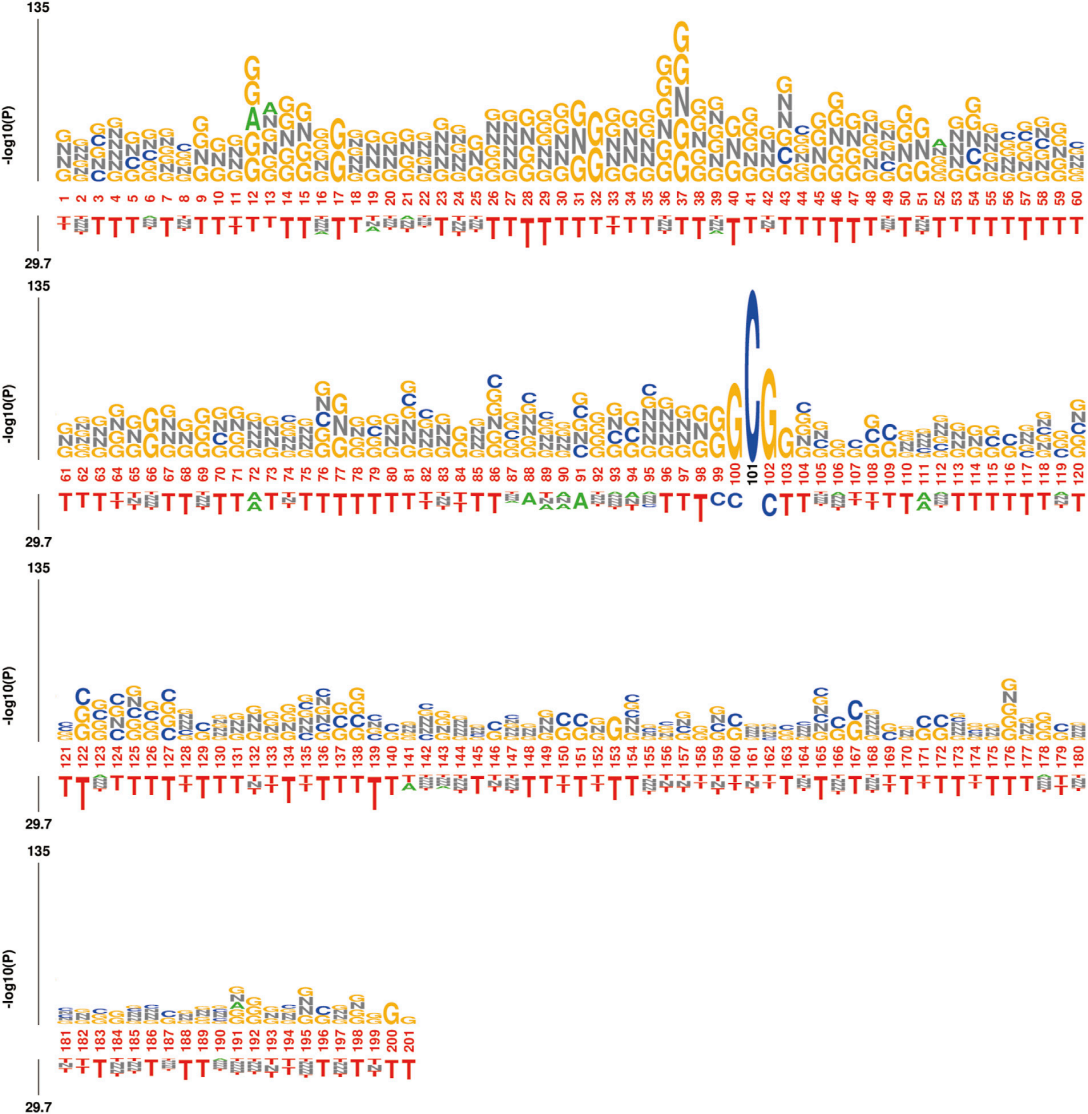
kpLogo: Visualization of k-mer Logo, displaying the most significant k-mers at each position on a 201 nt length sequence. The total height is scaled according to its *p*-value (log10 transformed) or test statistics, aiding in identifying enriched.

Furthermore, the trough phenomenon at the center point may be due to the construction method of the dataset, where both ac4C and non-ac4C sequences are centered around C, resulting in lower information content at that position. To further validate our observations, we analyzed the sequence using kpLogo, and the k-mer Logo (Figure 12) displayed the most significant k-mers at each position, scaled in height according to the *p*-value (log base 10 transformation), aiding in the identification of enriched motifs in the sequence. The height variation of the k-mers in Figure 12 is consistent with previous experimental results, showing a decreasing trend from left to right in the sequence and revealing significant enrichment of k-mers such as GNNG, GGNGG, and GNG between positions 20 and 60.

### 3.7 Robustness and generalizability of STM-ac4C

We collected two datasets from MetaAc4C, also originally derived from ac4C site data of human mRNAs obtained experimentally by Daniel Arango et al. The difference is that these datasets have been processed with CD-HIT, applying a sequence homology threshold of 0.4 to remove redundant sequences. Each sequence is 415 nucleotides (nt) long, with the ac4C modification site located at the center of the sequence. The first dataset is balanced, and the second is unbalanced, sharing the same independent test set. In the training set, the number of negative samples in the unbalanced dataset is five times that of the positive samples. The specific statistics of the two datasets are shown in Table 6. We trained the STM-ac4C on these two datasets and evaluated its performance metrics on the independent test set, with related results detailed in Tables 7 and 8. Other results in the tables are from MetaAc4C. Using a lower CD-HIT threshold can avoid overestimating the prediction model. Even on the balanced dataset processed with a 0.4 homology threshold by CD-HIT, the STM-ac4C model still demonstrated excellent performance, achieving the highest accuracy (Acc), Matthews correlation coefficient (MCC), and area under the curve (AUC) of 82.01, 64.02, and 88.04. On the unbalanced dataset, STM-ac4C achieved the highest MCC (66.64), indicating that STM-ac4C has advantages in dealing with unbalanced data. The above analysis indicates that STM-ac4C is robust and generalizable. However, compared with MetaAc4C, the AUC of STM-ac4C is 0.09% lower, and the sensitivity (Sn) is 2.14% lower, indicating there is still room for improvement of STM-ac4C in recognizing positive samples.

**TABLE 6:** Statistical overview of the balanced and unbalanced dataset.

Data types	Balance dataset		Unbalance dataset	
	Training	Testing	Training	Testing
Positive	1,148	467	1,148	467
Negative	1,148	467	5,439	467

**TABLE 7:** Performance evaluation over the balanced dataset.

Method	Sn (%)	Sp (%)	Acc (%)	MCC (%)	AUC (%)
PACES	8.12	**98.29**	53.21	14.84	53.22
XG-ac4C	58.23	94.43	76.33	56.5	76.33
DeepAc4C	**82.8**	75.58	79.19	58.57	86.49
MetaAc4C-noGAN	81.8	80.09	80.94	61.89	86.51
MetaAc4C	79.22	84.36	81.79	63.68	87.49
STM-ac4C	82.44	81.58	**82.01**	**64.02**	**88.04**

The bold font is used to distinctly indicate the highest values achieved for each evaluation metric.

**TABLE 8:** Performance evaluation over the unbalanced dataset.

Method	Sn (%)	Sp (%)	Acc (%)	MCC (%)	AUC (%)
PACES	7.28	**95.49**	79.76	4.87	51.39
XG-ac4C	12.63	95.35	80.6	12.8	53.99
DeepAc4C	79.65	81.37	80.51	61.03	86.4
MetaAc4C-noGAN	**81.56**	80.94	81.04	62.09	86.86
MetaAc4C	80.94	84.8	82.87	65.79	**89.51**
STM-ac4C	78.8	87.58	**83.19**	**66.64**	89.42

The bold font is used to distinctly indicate the highest values achieved for each evaluation metric.

## 4 Conclusion

This study introduces a novel deep learning model, STM-ac4C, designed to precisely identify N4-acetylcytidine (ac4C) modification sites on human mRNA. The model integrates selective kernel convolution (SKC), temporal convolutional networks (TCN), and multi-head self-attention mechanisms (MHSA), efficiently extracting and integrating multi-level features of RNA sequences for high-precision ac4C site prediction. SKC adapts to capture k-mer features of varying lengths, breaking the constraints of traditional fixed kernel sizes. TCN reveals long-term dependencies within mRNA sequences, enhancing contextual information and effectively learning temporal features. MHSA assigns weights to different features, achieving adaptive integration among them. For feature encoding, the study employs a one-hot encoding strategy to preserve the integrity of input sequences and minimize information loss. After ten-fold cross-validation and independent testing, STM-ac4C performs better in predicting ac4C sites, with key metrics such as accuracy, Matthew’s correlation coefficient, and area under the curve outperforming existing methods. Moreover, the model’s robustness and generalizability are validated on additional balanced and imbalanced datasets. Sequence region influence analysis by the model reveals key sequence features impacting ac4C site prediction, offering new perspectives and directions for future research. Nonetheless, STM-ac4C has certain limitations, such as not considering RNA secondary structures and other factors that may influence ac4C modifications nor being tested across different species or RNA modification types, limiting its generalizability in broader biological applications. Future work will further optimize the model, enhance the accuracy of ac4C site prediction, and extend its application to other species and RNA modification types while incorporating more features and data to explore broader application possibilities.

## Figures and Tables

**Table:** 

**ac4C**	N4-acetylcytidine
**rRNA**	ribosomal RNA
**tRNA**	transfer RNA
**snRNA**	small nuclear RNA
**RF**	random forest
**PSDSP**	position-specific dinucleotide sequence spectrum
**KNF**	k-nucleotide frequency
**XGboost**	extreme gradient boosting
**EIIP**	electron-ion interaction pseudopotentials
**PseEIIP**	electron-ion interaction pseudopotentials of trinucleotide
**CNN**	convolutional neural network
**GBDT**	gradient boosting decision tree
**NCP**	nucleotide chemical property
**ANF**	accumulated nucleotide frequency
**CNN**	convolutional neural network
**LSTM**	long short-term memory
**AutoML**	automated machine learning
**DenseNet**	densely connected convolutional networks
**Bi-LSTM**	bidirectional long short-term memory
**BERT**	bidirectional encoder representation from transformers
**WGAN-GP**	Wasserstein Generative Adversarial Nets - Gradient Penalty
**SOTA**	state-of-the-art
**SKC**	selective kernel convolution
**TCN**	temporal convolutional network
**MHSA**	multi-head self-attention mechanisms
**MLP**	multi-layer perceptron
**GAP**	global average pooling
**FC**	fully connected
**RNN**	recurrent neural networks
**DCNN**	deep convolutional neural networks
**Sn**	Sensitivity
**Sp**	Specificity
**Acc**	Accuracy
**MCC**	Matthews correlation coefficient
**AUROC/AUC**	area under the receiver operating characteristic curve
**ROC**	receiver operating characteristic

## Data Availability

The original contributions presented in the study are included in the article/Supplementary material, further inquiries can be directed to the corresponding authors.
